# Emergence of phylogenetically diverse and fluoroquinolone resistant *Salmonella* Enteritidis as a cause of invasive nontyphoidal *Salmonella* disease in Ghana

**DOI:** 10.1371/journal.pntd.0007485

**Published:** 2019-06-20

**Authors:** Cassandra Aldrich, Hassan Hartman, Nicholas Feasey, Marie Anne Chattaway, Denise Dekker, Hassan M. Al-Emran, Lesley Larkin, Jacquelyn McCormick, Nimako Sarpong, Simon Le Hello, Yaw Adu-Sarkodie, Ursula Panzner, Se Eun Park, Justin Im, Florian Marks, Jürgen May, Timothy J. Dallman, Daniel Eibach

**Affiliations:** 1 Department of Infectious Disease Epidemiology, Bernhard Nocht Institute for Tropical Medicine, Hamburg, Germany; 2 Division of Infectious Diseases and Tropical Medicine, Medical Center of the University of Munich (LMU), Munich, Germany; 3 National Infections Service, Public Health England, Colindale, United Kingdom; 4 Liverpool School of Tropical Medicine, Liverpool, United Kingdom; 5 Wellcome Trust Sanger Institute, Cambridge, United Kingdom; 6 German Centre for Infection Research (DZIF), Hamburg-Borstel-Luebeck, Germany; 7 Jessore University of Science and Technology, Jessore, Bangladesh; 8 Kumasi Centre for Collaborative Research in Tropical Medicine (KCCR), Kumasi, Ghana; 9 Institut Pasteur, French National Reference Center for Escherichia coli, Shigella and Salmonella, Paris, France; 10 Department of Clinical Microbiology, Kwame Nkrumah University of Science and Technology, Kumasi, Ghana; 11 International Vaccine Institute, Seoul, Republic of Korea; 12 Oxford University Clinical Research Unit, Ho Chi Minh City, Vietnam; 13 Department of Medicine, University of Cambridge, Cambridge, United Kingdom; University of Washington, UNITED STATES

## Abstract

**Background:**

*Salmonella enterica* serovar Enteritidis is a cause of both poultry- and egg-associated enterocolitis globally and bloodstream-invasive nontyphoidal *Salmonella* (iNTS) disease in sub-Saharan Africa (sSA). Distinct, multi-drug resistant genotypes associated with iNTS disease in sSA have recently been described, often requiring treatment with fluoroquinolone antibiotics. In industrialised countries, antimicrobial use in poultry production has led to frequent fluoroquinolone resistance amongst globally prevalent enterocolitis-associated lineages.

**Methodology/Principal findings:**

Twenty seven *S*. Enteritidis isolates from patients with iNTS disease and two poultry isolates, collected between 2007 and 2015 in the Ashanti region of Ghana, were whole-genome sequenced. These isolates, notable for a high rate of diminished ciprofloxacin susceptibility (DCS), were placed in the phyletic context of 1,067 sequences from the Public Health England (PHE) *S*. Enteritidis genome database to understand whether DCS was associated with African or globally-circulating clades of *S*. Enteritidis. Analysis showed four of the major *S*. Enteritidis clades were represented, two global and two African. All thirteen DCS isolates, containing a single *gyrA* mutation at codon 87, belonged to a global PT4-like clade responsible for epidemics of poultry-associated enterocolitis. Apart from two DCS isolates, which clustered with PHE isolates associated with travel to Spain and Brazil, the remaining DCS isolates, including one poultry isolate, belonged to two monophyletic clusters in which *gyrA* 87 mutations appear to have developed within the region.

**Conclusions/Significance:**

Extensive phylogenetic diversity is evident amongst iNTS disease-associated *S*. Enteritidis in Ghana. Antimicrobial resistance profiles differed by clade, highlighting the challenges of devising empirical sepsis guidelines. The detection of fluoroquinolone resistance in phyletically-related poultry and human isolates is of major concern and surveillance and control measures within the region’s burgeoning poultry industry are required to protect a human population at high risk of iNTS disease.

## Author summary

*Salmonella enterica* serovar Enteritidis is both a prominent global cause of zoonotic gastroenteritis, in association with the industrial production of eggs and poultry, and of bloodstream-invasive infection in sub-Saharan Africa, a clinical syndrome referred to as invasive nontyphoidal *Salmonella* (iNTS) disease. African epidemic iNTS strains are frequently multi-drug resistant, leaving fluoroquinolone antibiotics as key agents in reducing iNTS-associated morbidity and mortality. We analysed the genomes of *S*. Enteritidis collected in Ghana from patients with iNTS disease to investigate the emergence of fluoroquinolone resistance in the region. Extensive phylogenetic diversity was present, however, fluoroquinolone resistance was confined to a single clade causing global epidemics of poultry-associated gastroenteritis. This resistance has predominantly emerged locally, rather than being imported. We found that antimicrobial resistance patterns differed by *S*. Enteritidis clade, highlighting the challenges of devising empirical sepsis treatment guidelines in the absence of diagnostic microbiology facilities to monitor changes in resistance profiles. Furthermore, we detected fluoroquinolone resistance in closely related poultry and human isolates, suggesting a role of antimicrobial use within the growing local poultry industry in driving the emergence of resistance and a need for surveillance measures within this industry to protect a human population at high risk of iNTS disease.

## Introduction

Nontyphoidal *Salmonella* (NTS) are a leading cause of bloodstream infection in sub-Saharan Africa (sSA), a clinical syndrome referred to as invasive nontyphoidal *Salmonella* (iNTS) disease. iNTS disease in sSA is most commonly caused by *Salmonella enterica* serovars *Salmonella* Typhimurium or *Salmonella* Enteritidis, and predominantly affects children <3 years and adults with advanced HIV infection, with a case fatality rate of 19% [1]. The most recent global estimate for iNTS disease was 3.4 million cases in 2010, the majority of which (58%) were in sSA [2,3]. The lack of a human vaccine against NTS places a primary importance on interruption of transmission and the availability of effective antibiotics to reduce iNTS-related morbidity and mortality. The strains circulating in sSA are, however, frequently multidrug resistant (MDR; defined in this study as resistant to three or more antimicrobial classes), leaving third-generation cephalosporins and fluoroquinolones as the key agents in treating iNTS disease [4–6]. The emergence of both fluoroquinolone resistance mutations and extended spectrum beta-lactamase production amongst NTS in recent years could therefore lead to cases that are untreatable with currently available antibiotics in resource-poor settings [4,7]. Whilst the prevalence of fluoroquinolone resistance amongst African salmonellae is still relatively low in comparison to other regions, such as Asia, this is expected to change as these agents become more widely available [4,7–12].

There have been several reports of novel lineages of NTS associated with invasive disease in Africa [5,6,13,14]. A study of *S*. Enteritidis provided evidence for the recent emergence of two novel clades of this serovar, one geographically restricted to West Africa and the other to Central/East Africa [13]. Both clades differ substantially from the clade causing the global epidemic of poultry- and enterocolitis-associated *S*. Enteritidis. In common with *S*. Typhimurium multi-locus sequence type ST313 [5], the novel clades of *S*. Enteritidis both have distinct prophage repertoires, harbour an expanded multidrug-resistance plasmid and exhibit genomic degradation similar to that seen in host-restricted typhoidal salmonellae, which have a more invasive pathotype.

Although *Salmonella enterica* in Europe has historically remained susceptible to most antibiotics, resistance to ciprofloxacin is now significant (13.3% amongst human NTS isolates in 2015) and these figures are largely driven by *S*. Enteritidis [15] and consumption of poultry products contaminated with resistant strains [16,17]. Similarly, nalidixic acid (NA) resistance (a marker for diminished ciprofloxacin susceptibility, DCS), conferred by single point mutations in the *gyrA* gene at codons 83 or 87, has been found in as many as 50% of human *S*. Enteritidis isolates in European studies [16,18,19].

We recently reported a high prevalence of DCS amongst iNTS disease-associated isolates collected in the Ashanti region of Ghana between 2007 and 2012, particularly amongst *S*. Enteritidis, with 10/19 (53%) having reduced susceptibility compared to only 2% (3/129) of *S*. Typhimurium [20]. The underlying epidemiology of DCS amongst NTS strains in Africa remains poorly described. The increasing global trade in poultry and the high levels of ciprofloxacin resistance amongst NTS strains in food animals in high-income countries [15,21,22] support a possible role for importation of resistant strains. In particular, Ghana imports large volumes of live birds and poultry meat from abroad (https://comtrade.un.org). Alternatively, less regulated use of antibiotics in animal husbandry combined with a growth in intensive farming practises may promote the local emergence of resistance.

*S*. Enteritidis displays niche plasticity, with distinct clades that enable it to become a prominent cause of gastroenteritis globally in association with the industrial production of eggs and poultry, and of multidrug-resistant, bloodstream-invasive infection in Africa [13]. Ghana has both a human population at risk of iNTS disease and an expanding poultry industry. We therefore performed whole genome sequencing of iNTS disease-associated *S*. Enteritidis isolates from Ghana found to have a high rate of the DCS phenotype, as well as poultry isolates from the same region, in order to understand whether DCS was associated with African or global clades of *S*. Enteritidis, as control measures are likely to be different.

## Methods

### Study site and study population

Blood cultures were collected as part of hospital surveillance studies for bloodstream infections conducted between September 2007 and April 2015 at the Inpatient Department of Agogo Presbyterian Hospital and the Outpatient Department of St Michael’s Hospital, Pramso, both located in the Ashanti Region in central Ghana [10,20,23,24]. All children ≥30 days and ≤15 years of age presenting with either a temperature of ≥37.5°C or reported fever within the past 72 hours were enrolled in the study. One to three milliliters of blood was taken from each child following local antisepsis protocols and inoculated into a paediatric blood culture bottle (BACTEC Peds Plus/F, Becton Dickinson) and processed using a BACTEC 9050 blood culture system (Becton Dickinson). During 2010, recruitment took place and blood cultures were performed on the adult ward of the Agogo Presbyterian Hospital.

Both local and imported poultry meat was purchased between May to December 2015 from retailers and open markets within Kumasi, the capital of the Ashanti region. 15g of each meat sample was immediately placed in sterile homogeniser bags and transported refrigerated to the laboratory.

### Ethical approval and informed consent

Ethical approval was granted by The Committee on Human Research, Publications and Ethics, School of Medical Science, Kwame Nkrumah University of Science and Technology in Kumasi, Ghana (CHRPE/AP/427/13; CHRPE/101/09), the International Vaccine Institute, South Korea (IVI IRB#2008–002, 2011–001) and the Ethics Committee of the Medical Association Hamburg, Germany (PV4592). Written informed consent was obtained from adults or the parents or guardian of study children prior to enrolment.

### Laboratory procedures

Methods for the identification of *Salmonella* from blood culture have been described previously [20,24]. In brief, positive blood cultures were subcultured on Columbia blood agar, chocolate agar, and MacConkey agar (Oxoid, UK). *Salmonella* isolates were identified biochemically by API 20E tests (bioMérieux, France) and serotyped following the White–Kauffmann–Le Minor scheme. Ground meat samples were incubated overnight in Selenite broth (Oxoid) and subsequently cultured on Xylose Lysine Deoxycholate agar (Oxoid). Identification was performed as for blood culture isolates.

Ciprofloxacin minimum inhibitory concentrations (MICs) were determined by Etest (Oxoid) according to the European Committee for Antimicrobial Susceptibility Testing (EUCAST) guidelines [25]. Isolates were classed as ciprofloxacin susceptible (MIC ≤0.06 μg/mL), intermediate/diminished susceptibility (MIC >0.06 and <1 μg/mL) or resistant (MIC ≥1 μg/mL).

### Whole genome sequencing and bioinformatic analysis

DNA was extracted using the QIAamp DNA Mini-kit (Qiagen, Germany) according to the manufacturer’s instructions. Sequencing of extracted DNA from isolates 1–21 was performed by the Wellcome Trust Sanger Institute using the Nextera XT library preparation kit on the Illumina HiSeq 2000 (Illumina, USA) yielding 100bp paired-end reads. Extracted DNA from isolates 22–29 was sequenced by the Public Health England (PHE) Genome Sequencing Unit using the Nextera XT library preparation kit on the Illumina HiSeq 2500 (Illumina, USA) run in fast mode according to the manufacturer’s instructions, which yielded 2x 100bp paired end reads (see S1 Table for individual isolate details and accession numbers).

Multi-locus sequence typing (MLST) analysis was performed using MOST [26]. Identification of antimicrobial resistance determinants (ARD) was performed as previously described [27]. Quality trimmed Illumina reads were mapped to the *Salmonella enterica* Enteritidis reference genome P125109 (GenBank:AM933172) using BWA-MEM [28]. Single nucleotide polymorphisms (SNPs) were then identified using GATK2 [29] in unified genotyper mode. Core genome positions that had a high quality SNP (>90% consensus, minimum depth 10x, GQ> = 30) in at least one strain were extracted and RaxML v8.17 [30] used to derive the maximum likelihood phylogeny of the isolates under the GTRCAT model of evolution. Support for the maximum likelihood phylogeny was assessed via 100 bootstrap replicates.

Single linkage SNP clustering of the isolates within the PHE *S*. Enteritidis (eBurst Group 4) database, consisting primarily of clinical isolates from routine surveillance in the UK, but also sequences obtained from public databases, was performed as previously described [31]. FASTQ reads from all sequences in this study can be found at the PHE Pathogens BioProject at the National Center for Biotechnology Information (Accession PRJNA248792).

### Root-to-tip analysis

The temporal signal for a given phylogenetic tree was determined using TempEst v1.5.1 [32], using the tree and tabulated sample dates as input data.

### Plasmid analyses

The presence and absence of the MDR virulence plasmid, pSEN-BT (GenBank accession: LN879484), and a reference plasmid, pSENT (GenBank accession: HG970000), not associated with MDR, was determined by mapping (>90% consensus, minimum depth 10x, GQ> = 30) short reads from each isolate to the two plasmids using BWA-MEM [28] and plotting the depth coverage. A coverage threshold of > 90% was used to score the presence or absence of the virulence and reference plasmids. MDR was defined in this study as resistance to ≥ 3 antimicrobial classes.

## Results

### Isolates and sources

Twenty nine *S*. Enteritidis isolates were collected between 2007 and 2015; twenty five from children (median age 24 months, IQR 12–36 months), two from adults and two from poultry meat in 2015 (S1 Table). One poultry isolate originated from fresh slaughtered local meat while the other originated from imported (USA) frozen meat.

Of the 27 individual patients only one had a suspected admission diagnosis of gastroenteritis recorded (Table 1). The majority (21/27, 77.8%) had a non-focal febrile illness, such as malaria or sepsis, recorded.

**Table 1:** Clinical features and suspected diagnoses of patients (n = 27) at admission.

Clinical features/ suspected admission diagnosis		Number of patients (*%*)
Diarrhoea (reported at presentation)		5 (*18*.*5*)
Temperature <36 or >38°C (measured at admission)		20 (*74*.*1*)
Suspected admission diagnosis^a^	Non-focal febrile illness (including malaria, sepsis, enteric fever)	21 (*77*.*8*)
	Anaemia	9 (*33*.*3*)
	Gastroenteritis	1 (*3*.*7*)
	None recorded	5 (*18*.*5*)

^a^Patients could have more than one suspected admission diagnosis.

### Clades

**Fig 1:**
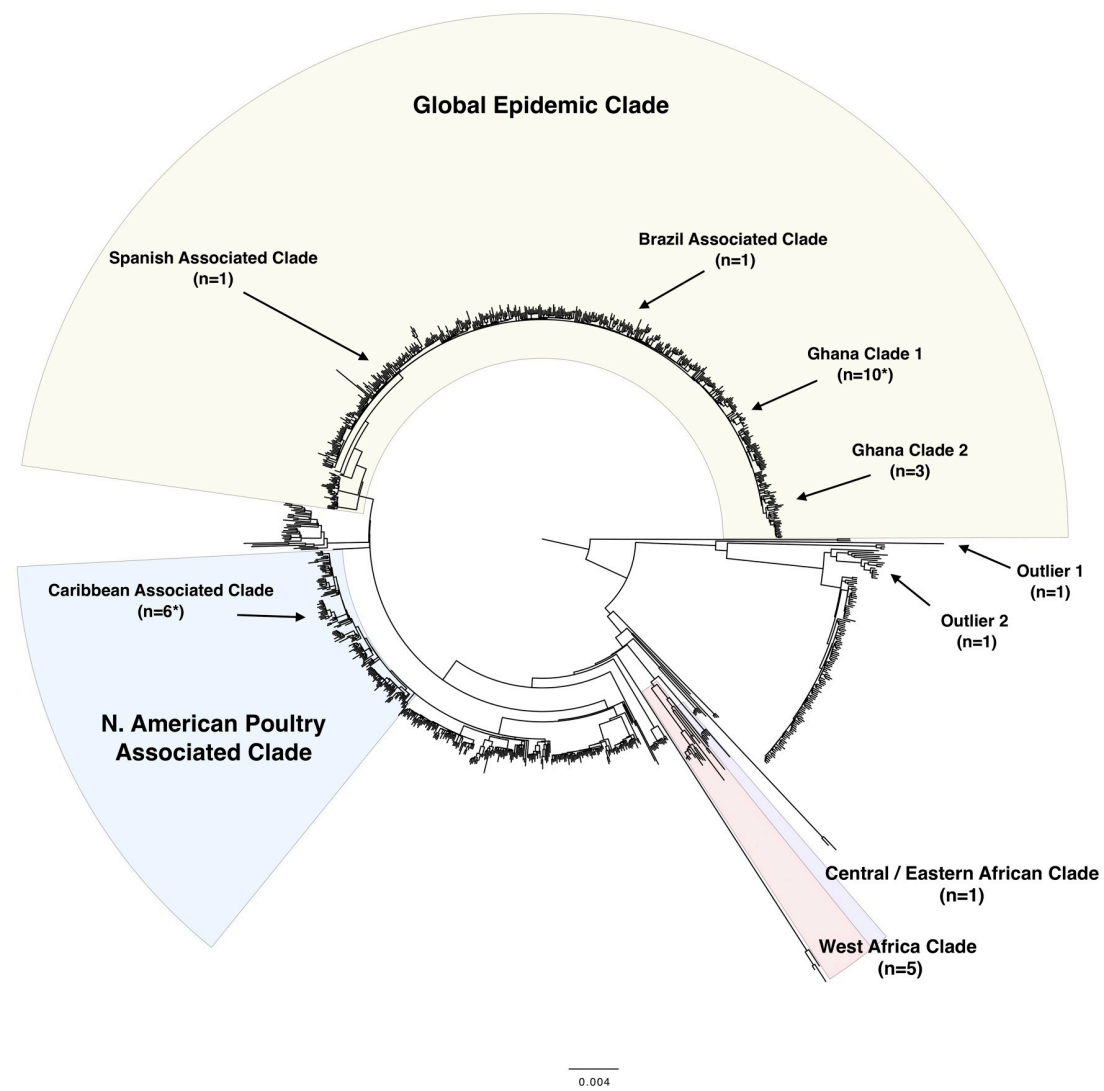
Midpoint-rooted maximum likelihood phylogeny of *S*. Enteritidis based on 1067 Public Health England surveillance isolates and the 29 Ghanaian isolates. The majority of the Ghanaian isolates fall into four major clades (a global epidemic clade, a second global clade containing a lineage associated with North American poultry, a Central/ East African clade and a West African clade). Within the global epidemic clade the Ghanaian isolates cluster into sub-clades. Numbers of Ghanaian isolates in each clade or sub-clade are noted in brackets (n =). An asterisk represents clades containing the poultry isolates (*). All clades are supported by bifurcating nodes with 100% bootstrap support. The scale bar shows nucleotide substitutions per site.

All Ghanaian isolates had Sequence Types (STs) within *S*. Enteritidis eBurst Group 4; 25/29 were ST11, and the remaining four were distinct single locus variants of ST11. When the isolates were placed in the context of the PHE surveillance isolates and a recently published global collection [13], a considerable amount of strain diversity was revealed (Fig 1). Fifteen isolates (14/27 human and 1/2 poultry) belonged to a ‘global epidemic clade’ containing isolates of multiple phage types (PT), including PT4 and PT1 [13], which have been linked to the global human epidemic of poultry-associated enterocolitis [33]. Six isolates (five human and one poultry) belonged to a lineage associated with shell egg outbreaks and egg-producing industries in North America [34]. Five human isolates belonged to the recently described West African clade and one human isolate clustered within the Central/East African clade, both associated with iNTS disease. The remaining two human isolates represented newly identified diversity in the *S*. Enteritidis population structure.

### Antimicrobial resistance determinant profiles

Thirteen isolates contained a single *gyrA* mutation at codon 87, corresponding with a DCS phenotype by Etest (MIC >0.06 and <1 μg/mL), and all belonged to the global epidemic clade. The majority of global epidemic clade isolates (13/15, 86.7%) contained the *gyrA* mutation, including the poultry isolate from this clade, which was obtained from a locally slaughtered bird. No other fluoroquinolone resistance determinants, including plasmid-mediated *qnr* genes, were detected amongst any of the isolates.

Apart from the *gyrA* 87 mutation, the most commonly identified resistance determinants were as follows: eleven isolates contained sulphonamide (most commonly sul-2), ten isolates streptomycin (strA and strB) and nine isolates tetracycline (most commonly tet(A)) resistance genes (S1 Table). Eleven isolates were MDR (10/27 human and 1/2 poultry). These comprised 7/15 (46.7%) of global epidemic clade isolates and 4/5 (80%) of West African clade isolates. The previously described MDR phenotype in African *S*. Enteritidis is conferred by an expanded MDR pSENT virulence plasmid (pSEN-BT) [13] of incFII/incFIB type. In this study, West African clade isolates harboured a different expanded MDR pSENT virulence plasmid of incompatibility type incI1.

### Global epidemic strains in Ghana

**Fig 2:**
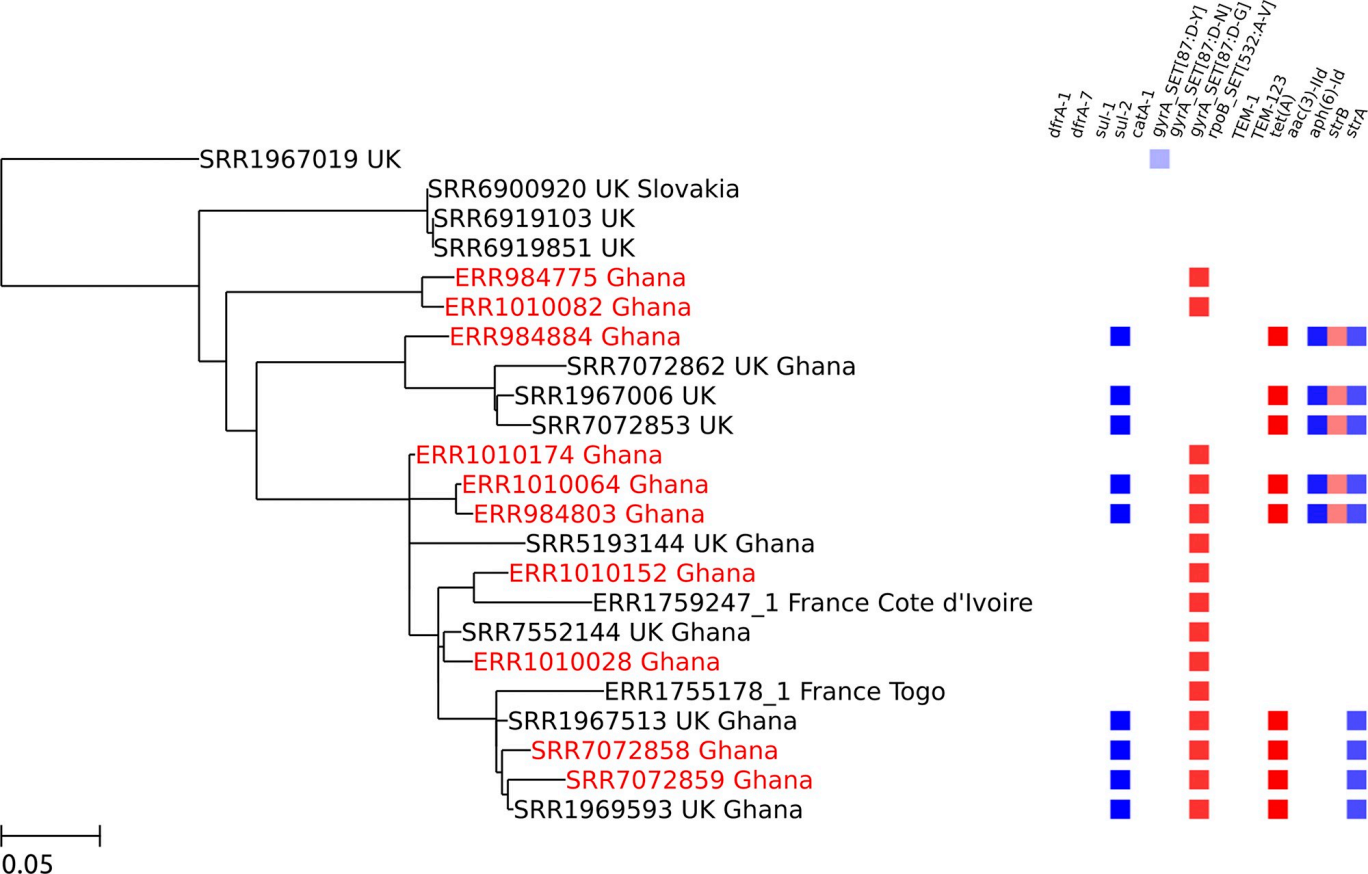
Zoomed-in maximum likelihood tree showing the largest monophyletic cluster of Ghanaian isolates within the global epidemic clade (Ghana clade 1). The ten Ghanaian isolates within this clade are highlighted in red (nine human bloodsteam isolates and one poultry isolate, SRR7072859). Each isolate in the tree is represented by its accession number followed by the country of origin. In the case of PHE isolates, second countries listed after the country of origin represent reported travel destinations. A heatmap showing the resistance genes present in each isolate (the *gyrA* 87:D-G mutation is shown as a red box) is displayed on the right. The scale bar shows nucleotide substitutions per site.

The majority (10/15) of the global epidemic clade isolates, including the poultry isolate (SRR7072859), clustered into a monophyletic clade with substantial diversity (max SNP distance of 92) (Ghana Clade 1 in Figs 1 and 2). The clade has a strong temporal correlation (root-to-tip R^2^ = 0.8) suggesting this clone has been present in Ghana for over twenty years. Clade 1 also contained ten isolates from human clinical cases from the UK, of which five reported recent travel to Ghana, as well as two isolates from human clinical cases from France. The French isolates were obtained in 2016 from stool samples from two children with gastroenteritis, returning from Ghana’s neighbouring countries, Togo and Cote D’Ivoire. Within Clade 1 9/10 Ghanaian isolates, 4/10 UK isolates and 2/2 French isolates harboured the same *gyrA* 87:D-G mutation. The tree topology reveals that this *gyrA* mutation likely evolved on two independent occasions within this region of West Africa (supported by 100% bootstrap values for these bifurcating nodes). Similarly, within this clade there have been multiple acquisitions of resistance determinants to sulphonamides, streptomycin and tetracycline.

**Fig 3:**
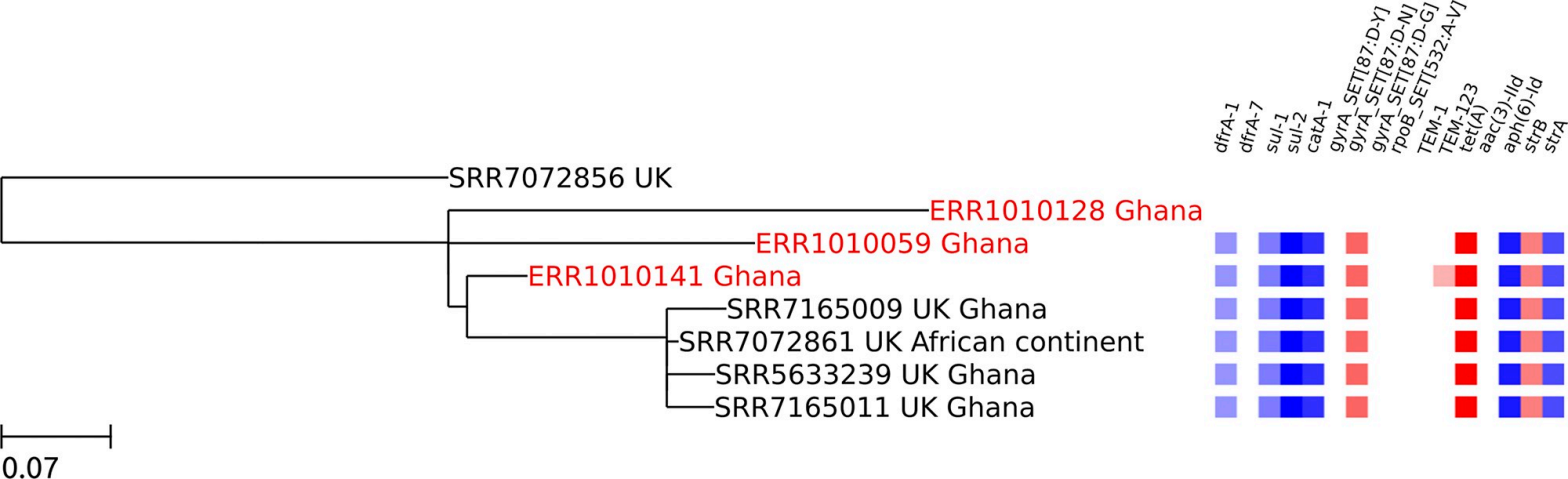
Zoomed-in maximum likelihood tree showing the smaller monophyletic cluster of Ghanaian isolates within the global epidemic clade (Ghana clade 2). The three Ghanaian human bloodstream isolates within this clade are highlighted in red. Each isolate in the tree is represented by its accession number followed by the country of origin. In the case of PHE isolates, second countries listed after the country of origin represent reported travel destinations. A heatmap showing the resistance genes present in each isolate (the *gyrA* 87:D-N mutation is shown as a pink box) is displayed on the right. The scale bar shows nucleotide substitutions per site.

A smaller monophyletic cluster within the global epidemic clade comprised three Ghanaian isolates and four UK clinical isolates (Ghana Clade 2 in Figs 1 and 3). Metadata associated with three of the UK isolates had travel to Ghana recorded. Six out of seven isolates contained the *gyrA* 87:D-N mutation and an IS6-flanked integron containing *catA-1*, *tet(A)*, *strA*, *strB*, *sul-2*, *sul-1* and *dfrA-1* genes. Within Clade 2, both emergence of the *gyrA* mutation and acquisition of the MDR locus has apparently occurred within the region (supported by 100% bootstrap values). One isolate (ERR1010141) had also acquired β-lactamase TEM-123.

**Fig 4:**
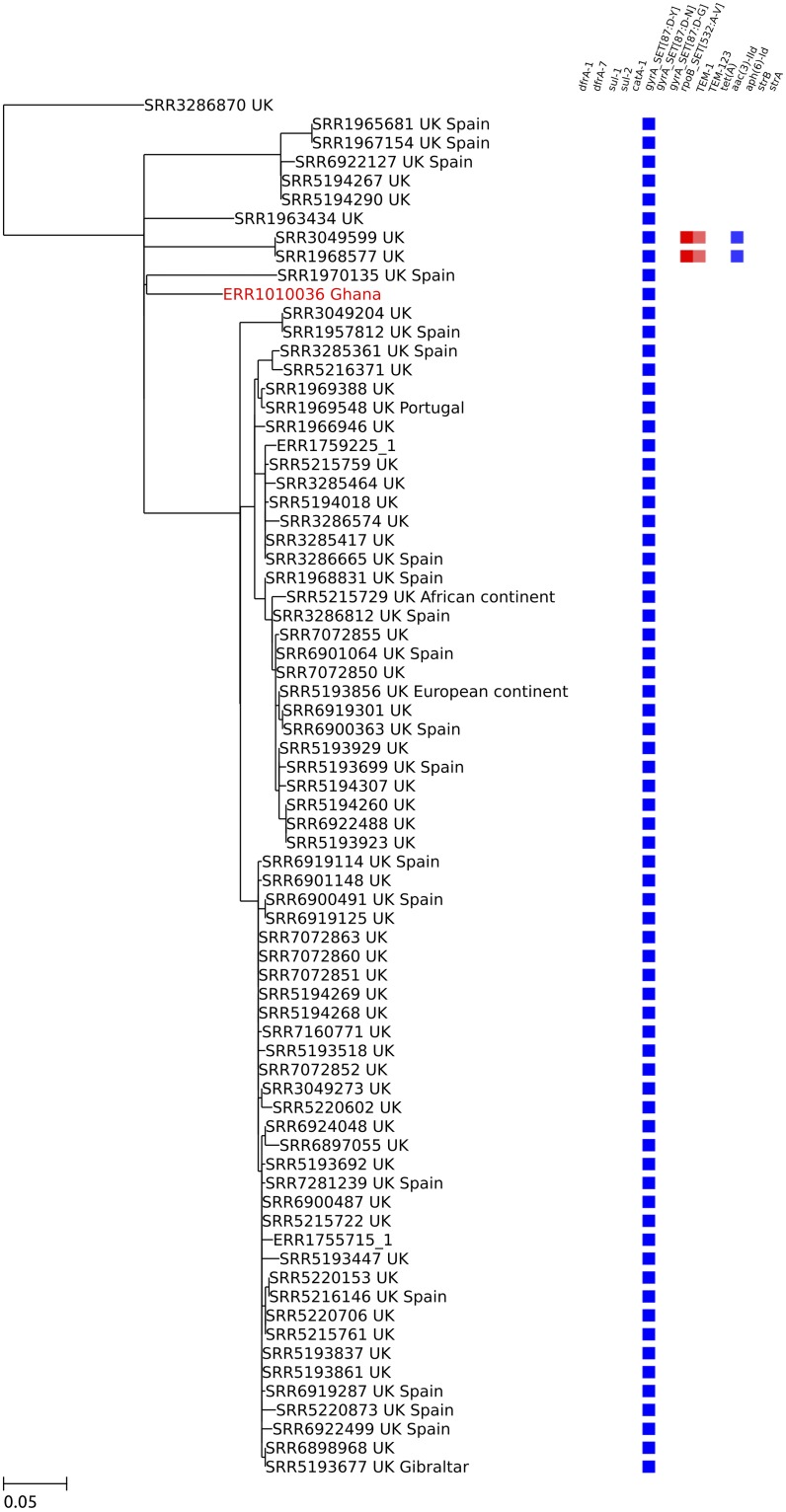
Zoomed-in maximum likelihood tree showing the Spanish travel-associated sub-clade of the global epidemic clade. One Ghanaian human bloodstream isolate was related to this sub-clade and is highlighted in red. Each isolate in the tree is represented by its accession number followed by the country of origin. In the case of PHE isolates, second countries listed after the country of origin represent reported travel destinations. A heatmap showing the resistance genes present in each isolate is displayed on the right. The *gyrA* 87:D-Y mutation (shown as a blue box) is present in every isolate of this sub-clade. The scale bar shows nucleotide substitutions per site.

A single Ghanaian isolate (ERR1010036), containing the *gyrA* 87:D-Y mutation, was found to be related (36 SNPs to closest isolate) to a large sub-clade of PHE isolates strongly associated with travel to Spain, all of which contained the same *gyrA* mutation (Fig 4). The remaining Ghanaian global epidemic clade isolate (ERR1010101) clustered with a PHE isolate associated with travel to Brazil (22 SNPs to PHE isolate), and both contained a *gyrA* 87:D-N mutation. No other resistance determinants were present in these two Ghanaian isolates.

### North American lineage

Six out of the twenty nine Ghanaian isolates, including one poultry isolate, clustered within a lineage historically associated with North American poultry and shell egg outbreaks [34] (Fig 1). Related PHE isolates were overwhelmingly associated with travel to the Caribbean while the Ghanaian poultry isolate was obtained from frozen meat imported from the USA. No antimicrobial resistance (AMR) determinants were identified in the six isolates. Half (4/8, 50%) of the isolates obtained in the last two years of sampling (2013–2015) belonged to this lineage.

## Discussion

We report extensive diversity amongst *S*. Enteritidis isolated from bloodstream infections in the Ashanti region of Ghana, with four recently described, major clades [13] represented and distinct clusters identifiable within a global poultry- and enterocolitis-associated epidemic clade. In this study, the majority (15/29, 52%) of isolates belonged to this global epidemic clade, whereas in other studies, African clades of *S*. Enteritidis have been the predominant cause of iNTS disease [12]. Despite the strong association between African clades and invasive disease [13], the considerable contribution of global clades to iNTS disease in this study is perhaps unsurprising given the importance of host risk factors in iNTS disease [35] and the recent expansion in intensive poultry farming in Ghana. It is also consistent with the experience in Malawi where both global and Central/ East African strains cause significant iNTS disease [13].

The diversity of AMR profiles between different clades of *Salmonella* and across sSA [this study, 12,13] highlights the challenges of implementing WHO IMCI/IMAI guidelines for the empirical management of sepsis and the importance of improving access to diagnostic microbiology facilities in low-income countries. In this study, almost half the global epidemic clade isolates were MDR (7/15, 47% compared to 4/5, 80% of West African clade isolates). The MDR phenotype has been shown to be strongly associated with the expansion of African NTS lineages and sequential iNTS epidemics, as well as the re-emergence of *S*. Typhi, in sSA [4,6,36]. Furthermore, MDR strains have been found to be strongly associated with NTS bacteremia compared to NTS diarrhoea [37]. The region may therefore be at risk of an iNTS epidemic due to MDR global strains.

Thirteen out of twenty nine (44.8%) isolates in this study contained a fluoroquinolone resistance mutation, specifically a *gyrA* 87 mutation, and all belonged to the global epidemic clade. This clade is transmitted through eggs and poultry, in settings with established industrialised poultry farming where fluoroquinolone resistance rates of around 10% are frequently observed amongst *S*. Enteritidis [15,22]. Currently, Ghana is both rapidly expanding its domestic poultry industry [38,39] and importing a significant quantity of live fowl and poultry meat from abroad (https://comtrade.un.org).

Our analysis of global epidemic clade isolates in this study provides evidence of both local emergence of *gyrA* 87 mutations and importation of DCS *S*. Enteritidis. A DCS isolate carrying the *gyrA* 87:D-Y mutation was related to a large sub-clade associated with travel to Spain in the PHE collection, raising the possibility that this strain was imported from Europe. 2.3 million kg of poultry meat was imported to Ghana from Spain in 2012 (https://comtrade.un.org). A further Ghanaian isolate, related to a PHE isolate with reported travel to Brazil, may also represent importation of the *gyrA* mutation. Brazil, along with the US and EU, exports large quantities of day old chicks and hatching eggs to Ghana [39].

The majority of DCS isolates however, appeared to originate from strains in which the mutation has likely emerged locally. The isolate from locally slaughtered poultry, containing the *gyrA* 87:D-G mutation, is related to a large monophyletic cluster of Ghanaian human bloodstream isolates. Within this cluster, the *gyrA* 87:D-G mutation appears to have evolved independently at least twice. These results are consistent with the emergence of the mutation in response to fluoroquinolone use in domestic poultry industries. Interestingly, two French isolates within the cluster were associated with travel to Togo and Cote D’Ivoire, which may represent trade in poultry between Ghana and its neighbours or wider circulation of the strain. A second cluster of Ghanaian isolates demonstrates *gyrA* 87:D-N mutation emergence and MDR locus acquisition within the region.

A recent cross-sectional survey found over 10% of poultry farms in the Ashanti region of Ghana, where the largest poultry farms in the country are located [39], reported fluoroquinolone use [40]. Indeed, the most common resistance determinants detected in the current study (other than the *gyrA* gene), conferring resistance to tetracyclines, sulphonamides and streptomycin, reflect the most commonly used antimicrobials in the Ghanaian poultry industry [40,41]. The global epidemic isolate from local poultry (both DCS and MDR) may therefore have originated from an intensive production background. The second poultry isolate (antimicrobial susceptible), which clustered within a lineage associated with North American poultry and shell egg outbreaks, was obtained from imported meat from the US, where broiler flock vaccination coverage is low [42].

Selection pressure arising from antimicrobial use within the region’s expanding poultry industry may be promoting the emergence of multidrug-resistant NTS disease-causing strains. The global epidemic of poultry- and egg-associated *S*. Enteritidis that began in the 1970s, and resulted in the collapse of national egg-producing industries, was eventually brought under control in European countries in the 2000s through a raft of farming hygiene measures as well as vaccination of poultry flocks [43]. The growing Ghanaian poultry industry may be facing the same challenges that the European industry faced during the 1980s and 1990s.

Our study is limited by its single site nature and analysis of a greater number of isolates from multiple sites would allow for a fuller understanding of *S*. Enteritidis dynamics in the region. Poultry sampling was conducted over a short period after completion of the hospital surveillance studies and only two isolates were obtained. However, both isolates clustered within monophyletic clades containing significant numbers of our bloodstream isolates, thus providing a link between poultry production and trade and human iNTS disease in the region.

Amongst global epidemic clade strains, a high rate of DCS may be developing in response to drug selection pressure within the region. Indeed, DCS strains that are likely circulating in Ghana’s expanding poultry industry appear to be responsible for a significant proportion of the *S*. Enteritidis invasive disease burden. Importantly, NTS disease and AMR patterns linked to poultry production are currently vaccine preventable problems, through a poultry rather than human vaccine. Surveillance and control measures within the burgeoning poultry industry, as well as a ‘one health’ approach, are required to protect a human population at high risk of iNTS disease.

## Supporting information

S1 Table Individual isolate metadata and antimicrobial resistance determinant profiles. (XLSX)
